# Fasciitis as a Complication of IgA Vasculitis

**DOI:** 10.7759/cureus.63344

**Published:** 2024-06-28

**Authors:** Kohichiroh Nii, Yuna Kondo, Natsumi Okamoto, Mayumi Yamamoto, Takashi Kusaka

**Affiliations:** 1 Pediatrics, Shodoshima Central Hospital, Shodoshima, JPN; 2 Postgraduate Clinical Training Center, Takamatsu Red Cross Hospital, Takamatsu, JPN; 3 Pediatrics, Faculty of Medicine, Kagawa University, Miki, JPN

**Keywords:** fascial vasuculitis, fasciitis, muscle involvement, myalgia, iga vasculitis

## Abstract

Immunoglobulin A vasculitis (IgAV) is a systemic small-vessel vasculitis caused by the deposition of IgA-based immune complexes, with myalgia being a rare complication. This study reports a pediatric case of IgAV with fasciitis. A five-year-old boy with no previous medical history was admitted to the hospital with abdominal pain and repeated bilious vomiting. Palpable purpura was observed on his face and right upper limb. Abdominal ultrasound and contrast-enhanced CT revealed decreased peristalsis and wall thickening of the fluid-filled duodenum, leading to a diagnosis of IgAV. Initial treatment with prednisolone and fasting improved his symptoms, but he complained of bilateral calf pain from day five with normal creatinine kinase levels. Fat-suppressed MRI on day 10 revealed high-signal areas around the soleus muscle, diagnosing fasciitis. Following steroid dose reduction, his myalgia worsened with difficulty falling asleep and the disability of standing up. Increasing the prednisolone dose alleviated his symptoms. The patient was discharged on day 23 without further myalgia. The pathogenesis of myalgia in IgAV remains unclear, but this case indicated a complication of fascial vasculitis and the effectiveness of steroid therapy. In conclusion, IgAV can be complicated by muscle involvement, and fasciitis should be considered a differential diagnosis of myalgia when creatinine kinase levels are normal. While supportive care is primary, steroid therapy should be considered depending on disease severity.

## Introduction

Immunoglobulin A vasculitis (IgAV), formerly called Henoch-Schönlein purpura, is a systemic small-vessel vasculitis caused by the deposition of IgA-based immune complexes. It commonly affects the skin, joints, gastrointestinal tract, and kidneys [1]. On the other hand, other organs, including the genitourinary, neurological, or pulmonary systems, have been reported [2]. Myalgia is a rare complication whose pathogenesis is not well understood. Myalgia is not well recognized in contrast to arthritic symptoms, especially in pediatric patients, because their complaints of physical symptoms are ambiguous. On the other hand, some patients complain of myalgia without creatinine kinase (CK) elevation, and there are reports of fasciitis among them in Japan [3]. Here, we report a pediatric case of IgAV with fasciitis.

## Case presentation

**Figure 1:**
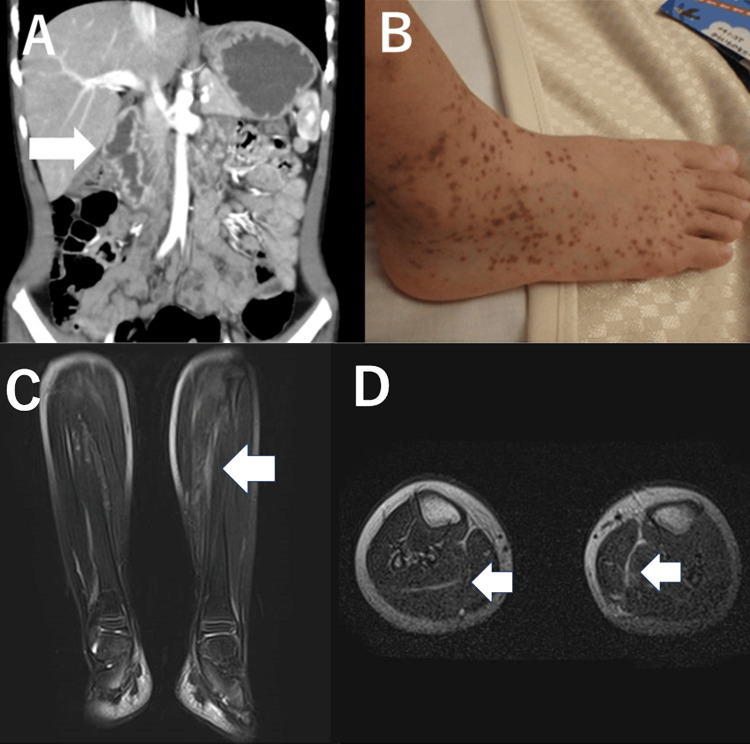
A. Abdominal contrast-enhanced computed tomography showing contrast enhancement of the duodenum with fluid retention. B. Palpable purpura on the leg. C and D. Fat-suppressed (chemical shift selective saturation (CHESS)) magnetic resonance imaging of the lower thigh shows high-signal areas around the soleus muscle.

A five-year-old boy with no previous history was admitted to our hospital for abdominal pain and repeated bilious vomiting. A few petechial purpuras were scattered on the face and right upper limb. He was afebrile and normotensive. Abdominal ultrasound showed decreased peristalsis and wall thickening of the duodenum, which was filled with fluid. Contrasted-enhanced computed tomography also showed contrast mainly in the descending part of the duodenum (Figure 1A). Laboratory findings (Table 1) revealed a C-reactive protein of 0.31 mg/dL (reference, <0.14 mg/dL), D-dimer of 3.3 µg/mL (<0.5 µg/mL), and factor XIII of 55.0% (<90%). Based on the European Alliance of Associations for Rheumatology (EULAR)/Pediatric Rheumatology International Trials Organisation (PRINTO)/Paediatric Rheumatology European Society (PRES) classification criteria, IgAV was diagnosed based on the palpable purpura (Figure 1B) and diffuse abdominal pain [1].

**Table 1:** The patient's laboratory data APTT: activated partial thromboplastin time; PT-INR: prothrombin time-international normalized ratio; PR3-ANCA: proteinase-3-anti-neutrophil cytoplasmic antibodies; MPO-ANCA: myeloperoxidase-anti-neutrophil cytoplasmic antibodies

Laboratory parameter	Value	Reference range
Complete blood count		
White blood cell	10600 (/µL)	4100 - 16300
Neutrophil	8162 (/µL)	1500 - 8000
Lymphocyte	1515 (/µL)	1500 - 7000
Eosinophil	74 (/µL)	0 - 740
Red blood cell	4.77×10^4^ (/µL)	410 - 540
Hemoglobin	12.6 (g/dL)	11.5 - 14.4
Platelet	47.1×10^4^ (/µL)	18 - 58.0
Coagulation factors		
APTT	24.2 (sec)	30 - 45
PT-INR	1.03	0.75 - 1.15
Fibrinogen	320 (mg/dL)	200 - 400
D-dimer	3.3 (µg/mL)	<0.5
Factor XIII	55%	>90
Blood chemistry		
Aspartate aminotransferase	21 (U/L)	17 - 39
Alanine aminotransferase	9 (U/L)	4 - 23
Lactate dehydrogenase	196 (U/L)	281 - 586
Creatine kinase	58 (U/L)	55 - 465
Total bilirubin	0.7 (mg/dL)	0.1 - 1.0
Urea nitrogen	17.4 (mg/dL)	6 - 20
Creatinine	0.34 (mg/dL)	0.3 - 0.7
Uric acid	10.8 (mg/dL)	3.7 - 7.0
C-reactive protein	0.31 (mg/dL)	<0.14
Total protein	7.3 (g/dL)	6.2 - 7.9
Albumin	4.3 (g/dL)	3.6 - 4.7
Triglyceride	139 (mg/dL)	30 - 86
Total cholesterol	189 (mg/dL)	125 - 230
Blood sugar	75 (mg/dL)	70 - 109
Annmonia	18 (µg/dL)	30 - 80
Ketone	5.2 (mmol/L)	<0.13
Immunology		
Immunoglobulin G	751 (mg/dL)	605 - 1460
Immunoglobulin A	182 (mg/dL)	36 - 221
Immunoglobulin M	70 (mg/dL)	80 - 322
50% hemolytic unit of complement	64 (U/ml)	25 - 48
Complement 3	146 (mg/dL)	84 - 151
Complement 4	38 (mg/dL)	17 - 40
Anti-streptolysin O	<5 (U/ml)	<240
Antinuclear antibody	negative	negative
Rheumatoid factor	<3 (mg/ml)	<15
PR3-ANCA	<1.0 (U/ml)	<10
MPO-ANCA	<1.0 (U/ml)	<20
Cryoglobulin	Negative	Negative
Urinalysis		
Protein	Negative	Negative
Occult blood	±	Negative
Glucose	Negative	Negative
Ketone	4+	Negative
Leukocyte esterase	Negative	Negative
Other		
Fecal occult blood	+	Negative

After admission, the patient was treated with 1 mg/kg/day of prednisolone and fasting, and his gastrointestinal symptoms improved within a day. Oral intake was slowly resumed after day three. He began to complain of bilateral calf pain while standing on day five. There was no swelling, redness, heat, tenderness, or limited range of motion in the knee or ankle joints, but there was pain and tenderness in the muscles around the gastrocnemius. Muscle involvement was suspected, but CK was not elevated. The myalgia gradually worsened after a dose reduction of prednisolone to 0.5 mg/kg/day on day 8. Fat-suppressed magnetic resonance imaging (MRI) on day 10 revealed high-signal areas around the soleus muscle (Figures 1C, 1D). Thus, fasciitis in these regions was diagnosed. C-reactive protein and D-dimer had temporarily improved after the initiation of steroid therapy but increased again to 1.38 mg/dL and 1.1 µg/mL, respectively. These findings indicated a relapse of IgAV. The myalgia was managed with acetaminophen, but he had difficulty falling asleep and a disability standing up; therefore, the dose of prednisolone was increased again to 1 mg/kg/day. This dose change was remarkably effective. Considering the course of relapse and the decrease in factor XIII, 40 units/kg/day of factor XIII were administered three times from day 16. The dose of prednisolone was tapered gradually, without any trouble, from day 18. The patient was discharged from the hospital on day 23. After discharge, the patient did not complain of myalgia and was followed up for nephritis.

## Discussion

Aside from the myalgia, the presence of palpable purpura, diffuse abdominal colicky pain, and the other clinical features of this case were typical of IgAV [1]. In a retrospective multicenter survey of adults in France, the complication rate of myalgia was reported to be about 6% [2]. There have been only seven reported cases of pediatric IgAV with myalgia [4-8]. In these cases, myalgia is often attributed to intramuscular hemorrhage. Watanabe et al. suggested another pathologic mechanism, namely, muscle ischemia due to necrotizing inflammation affecting the small vessels within skeletal muscle [7]. Fasciitis associated with IgAV, to date reported only in adult cases, has been attributed to leukocytoclastic vasculitis of intramuscular vessels [3]. Our patient did not undergo a biopsy, and the pathological changes are not known. Myalgia was present in the lower extremities without an increase in CK in all reported cases. Our literature search found no other cases of fasciitis in pediatric IgAV. Thus, reports of complications between IgAV and fasciitis are limited. However, Takahashi et al. reported a case of fascial vasculitis detected by MRI as a characteristic finding in a patient with microscopic polyangiitis (MPA) presenting with myalgia [9]. In this case of MPA, MRI showed high intensity around each muscular fascicle of the legs in the short T1 inversion recovery (STIR) image and the gadolinium-enhanced T1-weighted image. The pathological examination revealed necrotizing vasculitis localized in the deep fasciae and perimysium. Immunoglobulin A vasculitis also affects small vessels as well as MPA; thus, it can similarly cause necrotizing vasculitis in the fascia and show similar MRI findings. In summary, our patient was presumed to be affected by necrotizing vasculitis localized in the small vessels of fascia around the soleus muscle due to the deposition of IgA-based immune complexes.

Previous reports on pediatric IgAV have recommended supportive care for muscle involvement [8]. Non-steroidal anti-inflammatory drugs, such as acetaminophen, are important for pain management in addition to rest and exercise restrictions. Patients did not require additional treatment in those cases. In our case, however, supportive care alone was ineffective, and steroid therapy was necessary, with relapse occurring when the dose was reduced. As a result, the steroid dose had to be increased again. As in cases of gastrointestinal and joint involvement, steroid therapy was effective in reducing pain.

## Conclusions

Immunoglobulin A vasculitis may be complicated by muscle involvement, and fasciitis should be considered a differential diagnosis of myalgia in the absence of CK changes. Although supportive care is the basic treatment, steroid therapy is effective in reducing symptoms. Therefore, the use of steroids should not be uniformly recommended because of the risk of side effects; it should be considered depending on disease severity.

## References

[1] Ozen S, Pistorio A, Iusan SM (2010). EULAR/PRINTO/PRES criteria for Henoch-Schönlein purpura, childhood polyarteritis nodosa, childhood Wegener granulomatosis and childhood Takayasu arteritis: Ankara 2008. Part II: final classification criteria. Ann Rheum Dis.

[2] Audemard-Verger A, Terrier B, Dechartres A (2017). Characteristics and management of IgA vasculitis (Henoch-Schönlein) in adults: data from 260 patients included in a French multicenter retrospective survey. Arthritis Rheumatol.

[3] Himino A, Yoichiro H, Shintaro T, Hamada T (2020). Two cases of IgA vasculitis with fasciitis (Article in Japanese). Rinsho Hifuka.

[4] Allen DM, Diamond LK, Howell DA (1960). Anaphylactoid purpura in children (Schonlein-Henoch syndrome): review with a follow-up of the renal complications. AMA J Dis Child.

[5] Meadow R (1979). Schönlein-Henoch syndrome. Arch Dis Child.

[6] Somekh E, Fried D, Hanukoglu A (1983). Muscle involvement in Schönlein-Henoch syndrome. Arch Dis Child.

[7] Watanabe T, Abe Y (2004). Muscle involvement in a patient with Henoch-Schönlein purpura nephritis. Pediatr Nephrol.

[8] Kimura N, Ohnishi T, Sato S, Uejima Y, Suganuma E (2020). Immunoglobulin A vasculitis with intramuscular hemorrhage: a case report. Pediatr Int.

[9] Takahashi H, Tsuboi H, Abe S (2015). Magnetic resonance imaging can reveal fascial vasculitis in a patient with microscopic polyangiitis. Scand J Rheumatol.

